# Effect of Furniture Weight on Carrying, Lifting, and Turning of Chairs and Desks among Elementary School Children

**DOI:** 10.1371/journal.pone.0128843

**Published:** 2015-06-08

**Authors:** Lu’lu’ Purwaningrum, Kyotaro Funatsu, Jinghong Xiong, Cucuk Nur Rosyidi, Satoshi Muraki

**Affiliations:** 1 Human Science International Course, Graduate School of Design, Kyushu University, Fukuoka, Japan; 2 Department of Interior Design, Faculty of Arts and Design, Sebelas Maret University, Surakarta, Indonesia; 3 Faculty of Sports Science, Kyushu Kyoritsu University, Fukuoka, Japan; 4 Department of Industrial Engineering, Faculty of Engineering, Sebelas Maret University, Surakarta, Indonesia; 5 Department of Human Science, Faculty of Design, Kyushu University, Fukuoka, Japan; Örebro University, SWEDEN

## Abstract

Rearranging furniture in elementary school classrooms encourages classroom activities. In elementary schools in Indonesia and some other developing countries, usually only one style of furniture is used for all children, and the furniture is heavy and oversized for younger children. This affects their ability to carry it. The objective of this study is to investigate the effects of elementary school furniture weight and children’s age on performance of three carrying tasks (carrying a chair, lifting and turning a chair on a desk, and carrying both a chair and a desk together), from the ergonomics point of view. A total of 42 schoolchildren (ages 6–9; 17 Indonesian, 25 Japanese) participated in this study. Two types of Japanese chairs (Chair A and B, weight: 3.2 kg and 3.9 kg), one type of Indonesian chair (Chair C, weight: 5.0 kg), and two types of desks (height: 58 cm and 68 cm) were used. Indonesian chairs took significantly longer time to carry than the two Japanese chairs, and there was a significant negative relationship between age and task time for Chairs B and C, but not Chair A. Success rates for lifting and turning the chair declined as age decreased and chair weight increased, but were not significantly influenced by desk height. Success rates for carrying a chair and desk together significantly decreased with heavier furniture. Children aged six showed an extremely low success rate in almost all conditions. In conclusion, children’s ability to carry furniture is affected by their age and furniture characteristics, especially weight. In order to encourage classroom activities in elementary school, school furniture should be of appropriate weight. Supervision for younger children is required during classroom furniture arrangement.

## Introduction

It has long been noted that elementary schools should provide desks and chairs that are easy for students to move and carry. School furniture (e.g., chairs and desks) that is easy to move and carry could help to improve the quality of education [1–4]. Previous studies have suggested that seating arrangements should be changed on a regular basis, because they may have a significant influence on students’ behaviors [1–3]. For example, furniture in rows and columns may lead students to ask more questions, whereas semi-circle configurations may encourage social interaction among students [1]. Considering the limited carrying and lifting capabilities of children, and that lighter or proper-weight furniture requires less effort to move, use of this type of furniture could save time as well as reduce the risk of injury.

Increasing the weight that one carries increases the physiological cost of walking, and the load on leg muscles [5]. Studies have suggested that the weight of backpacks carried by children should be proportional to their body weight [6–8]. The body weight of children normally increases with age [9–11]. Therefore, the weight of school furniture should correspond to children’s ages and body weights.

Some solutions have been proposed with regard to this issue. Breithecker [4] introduced a new swivel chair design using castors, named the “ergo dynamic” or “movement ergonomic” chair. This chair can be easily moved by children in the classroom [4]. In addition, the attention and concentration of students using these chairs have been shown to improve by over 70%, compared with students using traditional chairs [4]. Although this design provides an easy-to-move chair, developing countries have difficulty implementing this change, because of budgetary concerns and limited manufacturing techniques.

**Fig 1:**
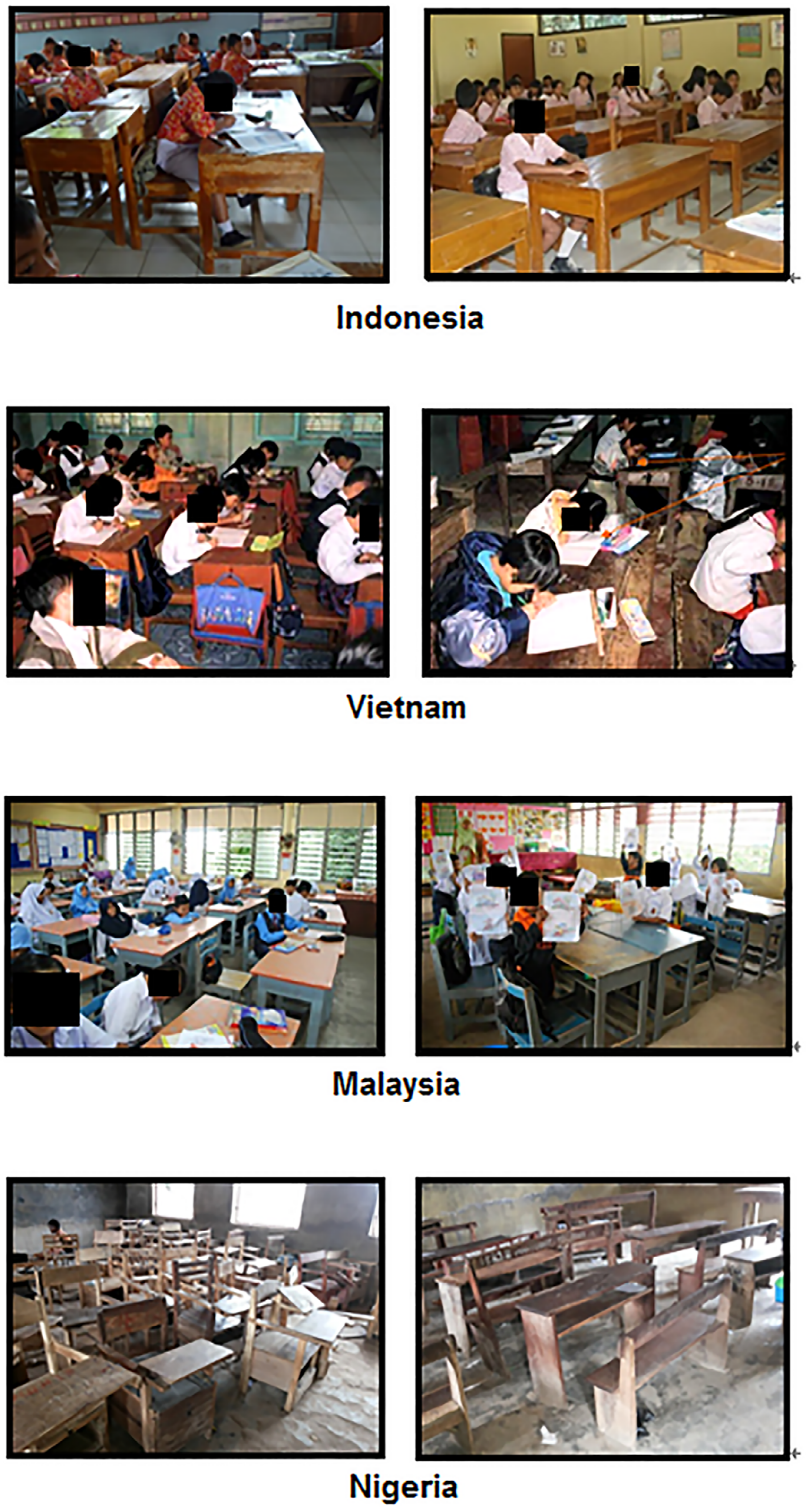
Chairs and desks of Australian, Japanese, and Scottish elementary schools.

In developed countries such as Australia, Japan, and Scotland, elementary school chairs and desks are lighter, and may be easily carried and moved by all children (Fig 1); however, weight guidelines still need to be better applied prior to manufacturing. Many researchers have investigated appropriate furniture for elementary school children in their respective countries [12–16], but these studies have not focused on weight issues. In Japan, school furniture dimensions are regulated based on ISO 5970 [17], so that furniture appropriate for children of certain body dimensions and age ranges is produced. However, weight guidelines are needed for manufacturing.

**Fig 2:**
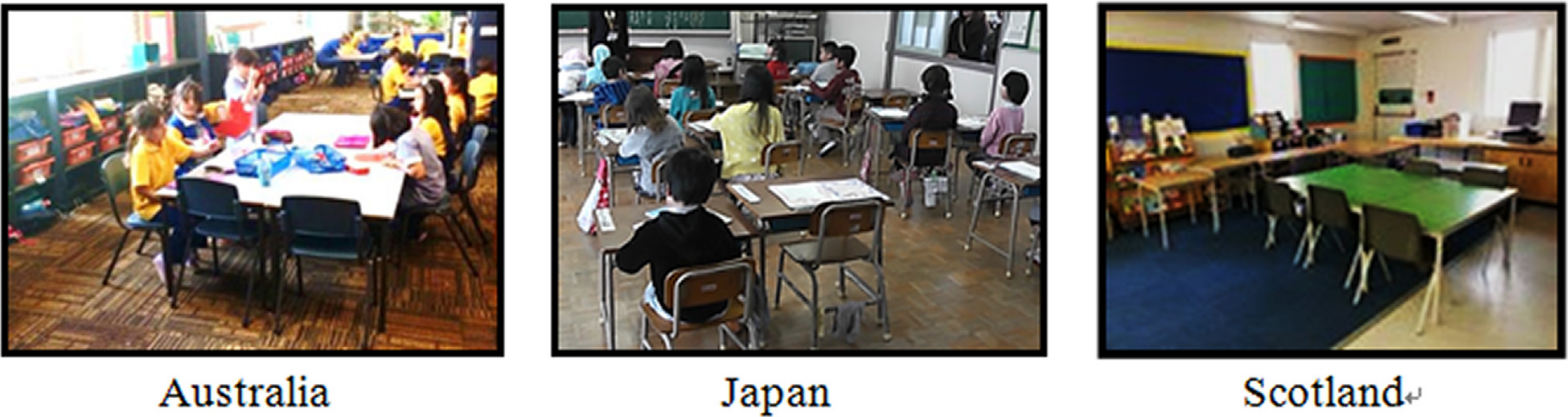
Chairs and desks of elementary schools in Indonesia, Vietnam, Malaysia and Nigeria.

In developing countries such as Nigeria, Malaysia, Indonesia, and Vietnam (Fig 2), the weight of elementary school furniture has not been sufficiently considered. Since there are insufficient studies on furniture weight, children may carry chairs and desks that are too heavy for them. In Indonesia, the Ministry of National Education has a regulation that elementary school chairs must be easily carried by children [18]. In addition, the desired dimensions (including weight) of school furniture differ between grades 1–3 and grades 4–6 [18]. However, in most developing countries, across every age and grade in elementary schools, chairs have uniform dimensional standards [19–21] This may discourage movement of chairs and desks for classroom activities.

Taking all of the above finding into account, it is necessary to propose a weight guideline for elementary school furniture that is proportional to children’s body dimensions at certain ages. The present study investigated the effects of elementary school furniture weight and children’s age on performance of three tasks—carrying a chair, carrying both a chair and a desk, and lifting a chair onto a desk. This study also compared the difficulty of these tasks for Indonesian and Japanese furniture.

## Materials and Methods

### Participants

Healthy Indonesian and Japanese children (N = 42) who could understand the experimenters’ instructions participated in this study. The Indonesian children included 6 boys and 11 girls, and the Japanese children included 12 boys and 13 girls. Their ages ranged between 6.0 and 9.9 years and they were categorized into four age-range groups: 6 (6.0–6.9), 7 (7.0–7.9), 8 (8.0–8.9), and 9 (9–9.9) years. In both Indonesia and Japan, children go to elementary school for six years, starting at age 6. The age range of our participants corresponded to the lower and middle grades (first to fourth year) of elementary school. Table 1 presents the number of participants by age-range group, gender, and nationality.

**Table 1:** Number of participants.

	Indonesian (N)		Japanese (N)	
Age group (Age range, years)	Boys	Girls	Boys	Girls
6 (6–6.9)	1	2	5	1
7 (7–7.9)	1	3	4	2
8 (8–8.9)	3	4	3	7
9 (9–9.9)	1	2	0	3
Total	6	11	12	13

Before the experiment, participants’ parents signed informed consent forms. This study was approved by the ethics committee of the Faculty of Design, Kyushu University, Japan (Approval Number: 125).

### Experimental instruments

Representative furniture from Indonesian and Japanese public elementary schools were used as the main equipment in this experiment, including one Indonesian chair, two Japanese chairs, and one Japanese desk.

#### Chairs

**Fig 3:**
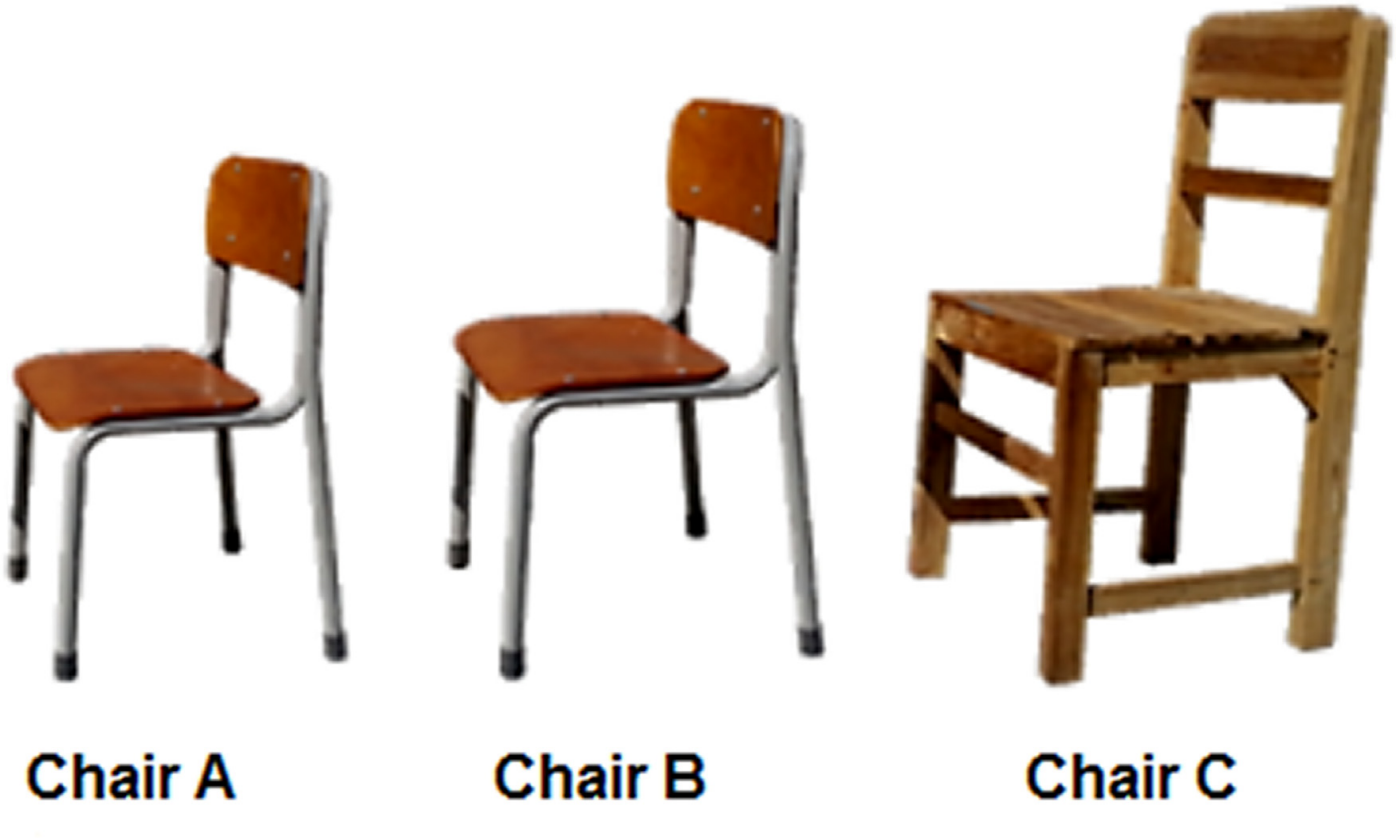
Chairs for the experiments. Chairs A and B are Japanese elementary school chairs; Chair C is a typical Indonesian elementary school chair.

Two Japanese (Chairs A and B) and one Indonesian (Chair C) chairs were used to create three different chair weight conditions (Table 2). In Japan, some sizes of chairs and desks (proportional to student height) based on ISO 5970 [17] are most readily available in elementary schools. This experiment employed two sizes of Japanese chairs. Chairs A and B were Size 2 (340 mm width x 290 mm depth x 300 mm seat height) and Size 3 (360 mm weidth x 290 mm depth x 340 mm seat height), respectively (Fig 3). Chairs A and B were appropriate for students whose height ranged between 117 and 130 cm, and between 131 and 144 cm, respectively. Although the material was the same for both chairs, the weights were different due to the size difference (Chair A: 3.2 kg, Chair B: 3.9 kg).

**Table 2:** Dimensions of Indonesian and Japanese chairs.

Dimension	Chair		
	A	B	C
Seat height^a^ (mm)	300	340	420
Weight (kg)	3.2	3.9	5.0

^a^Distance from the seat to the bottom of the chair.

In contrast, in Indonesia, there is no precise standard for school furniture design for each age. Accordingly, the ordinary elementary school chair had a seat height and weight of 420 mm and 5 kg, respectively (Table 2). This is a typical chair used in most public Indonesian elementary schools [18, 22, 23]. According to our survey regarding chair weights in 11 public Indonesian elementary schools (prior to this experiment) [23], 5 kg was among the lowest range of chair weights in Indonesian elementary schools. The seat height of the chair was in the same range as chairs generally used in Indonesian elementary schools (380–450 mm) [18, 23].

#### Desks

**Fig 4:**
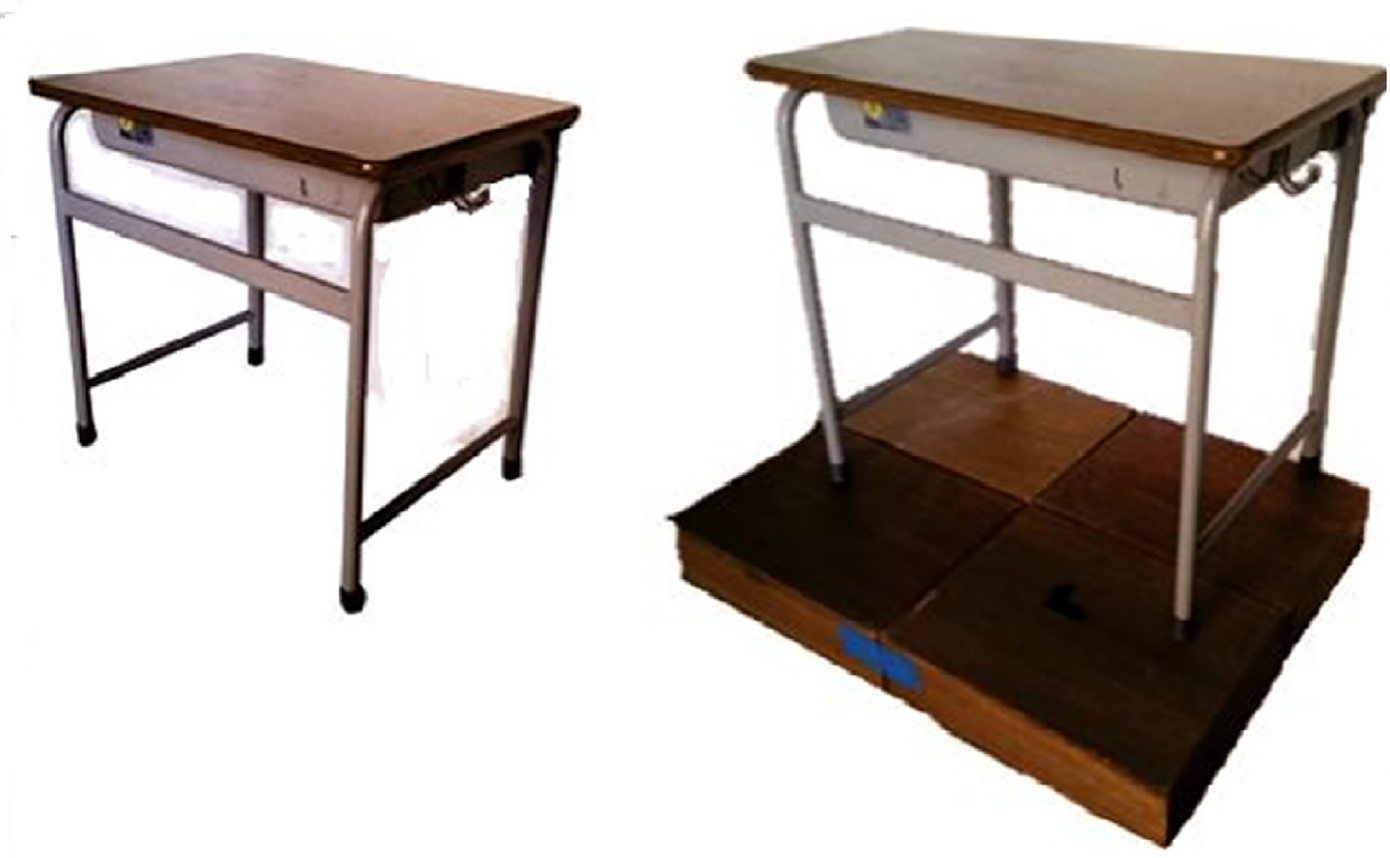
The Japanese desk without additional height (left) and with additional height (right).

Only a Japanese elementary school desk was used in this experiment (Fig 4). This desk was for children whose height ranged from 131 to 144 cm (Size No. 3 of Japanese elementary school desk), and met ISO 5970 [17]. The height and weight of the desk were 580 mm and 8.4 kg, respectively (Table 3). The Indonesian desk was simulated using the Japanese desk with additional height and weight. The height and weight of the desk was based on the typical Indonesian elementary school desk, which was 55–75 cm [18, 23]. In lifting and turning the chair, additional height was added using a box (height: 100 mm) (Fig 4). This was an adequate simulation of an Indonesian desk for this task, because the main goal was to investigate the effect of the chair weight. In carrying a chair and desk together, additional weight created by placing 4.6 kg of iron in the desk drawer was adequate to simulate the heavier Indonesian desk.

**Table 3:** Weights for various combinations of carrying both a chair and a desk.

Furniture	Combination			
	A	B	C	D
Chair (kg)	3.2	3.9	5.0	5.0
Desk (kg)	8.4	8.4	8.4	8.4
Additional weight (kg)	-	-	-	4.6
Total weight (kg)	11.6	12.3	13.4	18.0

### Experimental tasks, conditions, and procedures

The experiment was conducted in a large flat space over six days in February and March 2013. Participants performed three different tasks: 1) carrying a chair, 2) turning and lifting a chair on a desk, and 3) carrying both a chair and a desk together. Before each task, we gave participants brief instructions about how to complete each task, and time for practice. To prevent injury, participants wore non-slip work gloves and shoes. In addition, we instructed them to stop the task immediately if they felt it was impossible or dangerous to continue. During the task, an adult stood beside the participants for safety.

Participants performed tasks from lighter/smaller to heavier/larger furniture, in order to prevent injury, and to allow participants to judge easily whether they could perform the next task level. After each task, an experimenter ensured that factors such as pain, fatigue, lack of motivation, etc. would not influence performance of the next condition. During each task, participants’ movements were recorded using a digital video camera (Panasonic, HC—V 300 M, Japan) at 30 frames per second.

#### Task 1: Carrying a chair (Fig 5)

This task involved bringing each chair (A, B, and C) from the start to finish line (a 3 m distance). A chair was placed sideways behind the start line, and participants sat on the chair to wait for the start command. After we gave a signal to start, they stood up from the chair, raised the chair with their hands, carried it toward the finish line, and put it down on the floor beyond the finish line. Before tasks, we instructed them that they 1) could hold any part of the chair, 2) should not push or slide the chair on the floor, and 3) should walk at an ordinary speed during the task.

**Fig 5:**
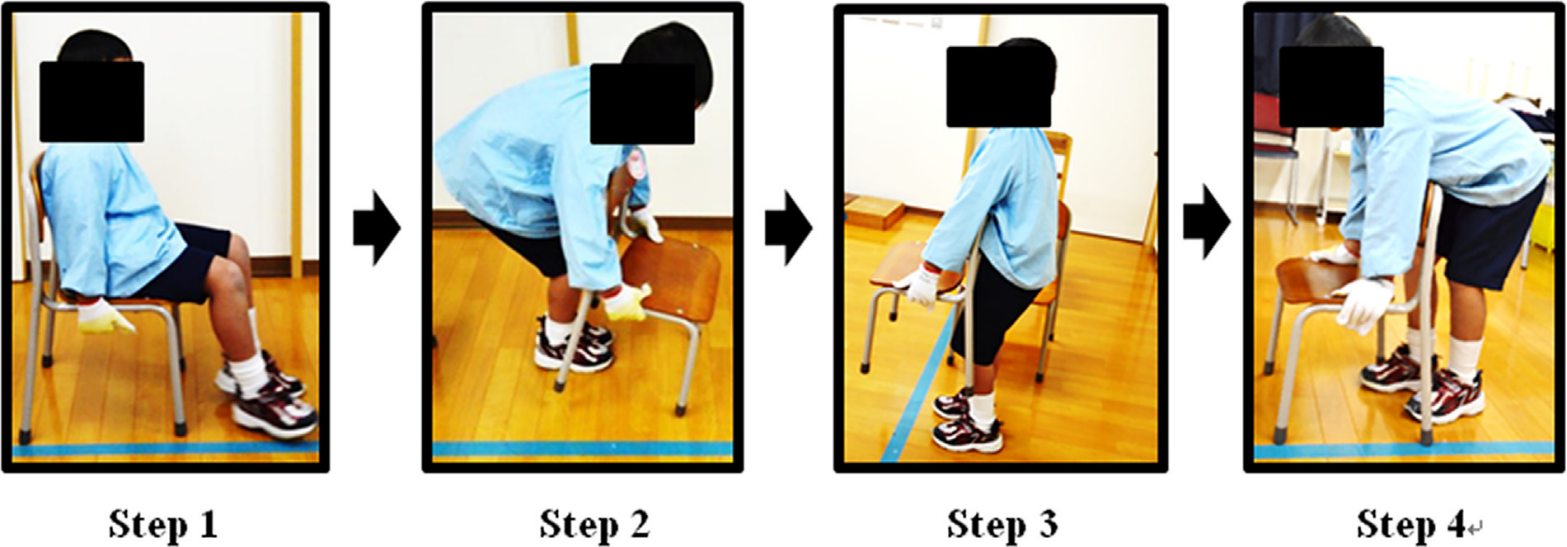
Task 1: Carrying a chair a distance of 3 m. Step 1: start position; Step 2: standing up (start time); Step 3: carrying the chair; Step 4: putting the chair down (finish time).

#### Task 2: Lifting and turning a chair upside down

This task involved turning a chair upside down and putting it on a desk. In this task, three chair weights (Chairs A, B, and C) and two desk heights (standard desk height: 580 mm, higher desk height: 680 mm) were employed.

**Fig 6:**
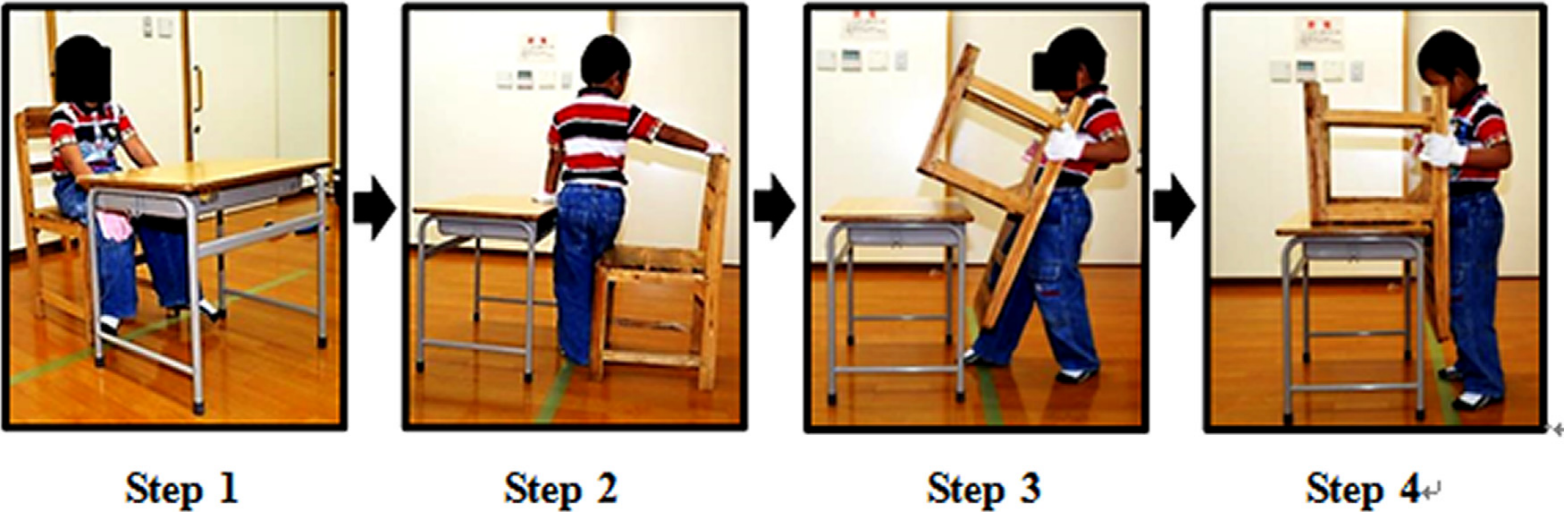
Task 2: Turning a chair upside down. Step 1: starting position; Step 2: standing up (start time); Step 3: turning the chair upside down; Step 4: putting the chair on the desk (finish time).

A chair was placed in front of the desk as indicated in Fig 6. First, the participant sat on the chair to wait for the start command. After the start command, the participant stood up from the chair, turned the chair upside down, and put it on top of the desk, as shown in Fig 6. Participants could hold any part of the chair, and they could rotate the chair on the floor if necessary.

#### Task 3: Carrying both a chair and a desk

**Fig 7:**
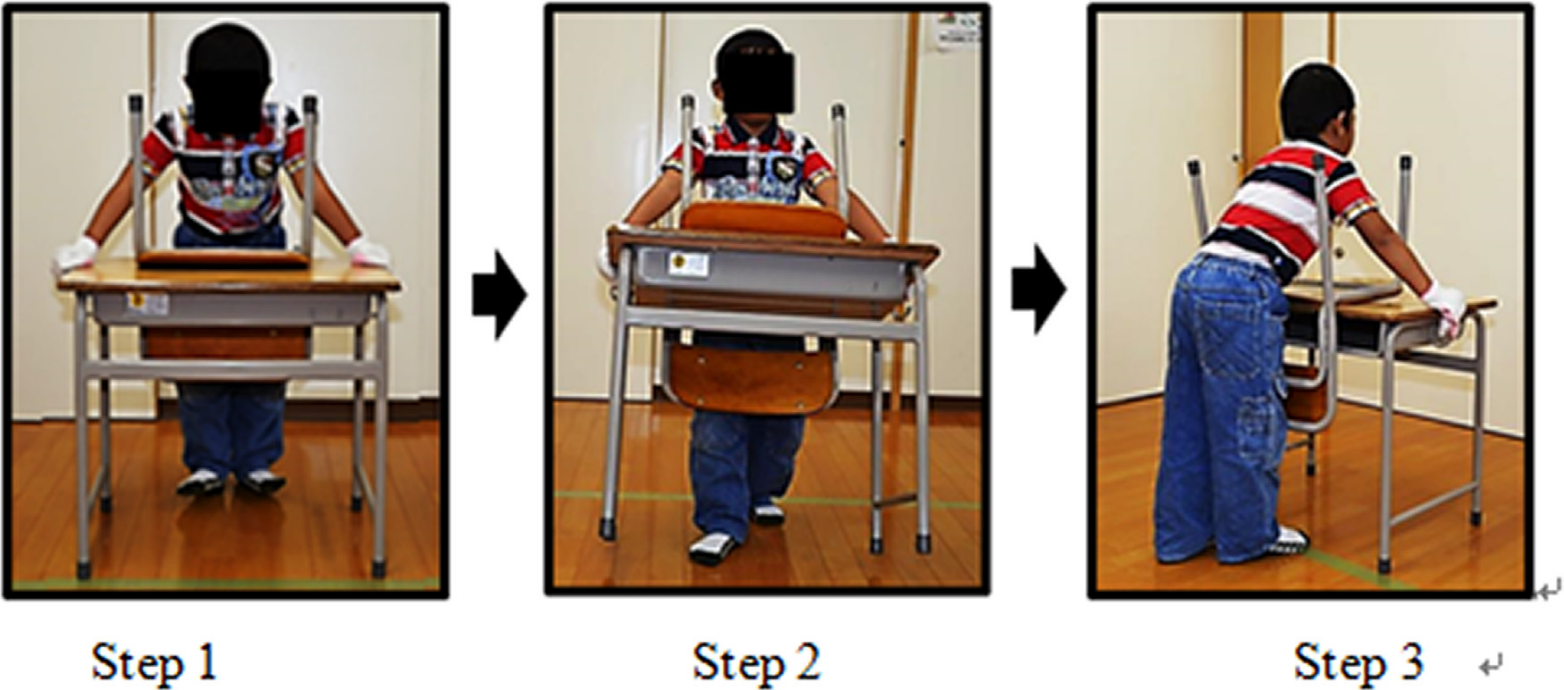
Task 3: Moving a chair and a desk together. Step 1: Starting position (start time); Step 2: carrying; Step 3: placing on the floor (finish time).

This task involved moving a desk with a chair, which was placed upside down on top of the desk (Fig 7), from the start to finish line (a 3-m distance). In this task, four conditions of different total weight (11.6, 12.3, 13.4, and 18 kg) were employed using three chairs (Chairs A, B, and C), a desk, and additional weight as specified previously (Table 3).

A desk was placed facing forward behind the start line. First, the participant stood in front of the desk, and held the sides of the desk top with both hands while waiting for the start command. After the start command, participants carried the desk and chair to the finish line, and placed it on the floor beyond the finish line. Before the task, participants were instructed to walk at a normal speed and to push or slide the desk across the floor.

### Measurements

#### Physical characteristics of participants

Before the experiment, height, weight, body mass index (BMI), and grip strength were measured. Grip strength was measured for both hands using a digital handgrip dynamometer (Takeikiki, A 5401, Japan).

#### Task time

The time taken to complete each task (task time) was measured using frame-by-frame playback of the recorded video. In Task 1, the start time was the moment when one chair leg bottom was raised off the floor, and finish time was when one chair leg bottom touched the floor. For Task 2, the start time was when one chair leg bottom was lifted, and the finish time was when the chair’s seat was placed on the table. Lastly, for Task 3, the start time was when one leg bottom of the desk and chair was raised, and finish time was when one was placed on the floor.

#### Successful and unsuccessful lifting and turning of the chair

In the task involving lifting and turning a chair onto the desk, we judged that children were successful if they could complete the task without dropping the chair, without stopping for a while in the middle of the task, and without putting themselves in danger. Dangerous situations were identified as those in which children carried the chair with an unstable grip and they were at risk of dropping it.

### Statistical Analysis

Statistical analyses were performed using IBM SPSS Version 21.0 (2012) for Windows, Chicago, USA. Descriptive results were presented as means and standard deviations. Two-way analysis of variance (ANOVA) was used to identify differences in physical characteristics related to gender and nationality. Pearson correlation coefficients were calculated to analyze the relationship among children’s physical characteristics, between children’s physical characteristics and task time, and between chair type and task time. A repeated measures one-way ANOVA was employed to compare task time of the three chair types in the chair-moving task, followed by post-hoc pairwise Bonferroni-corrected comparison tests for significant differences among chair types. The exact chi-square test was used to compare success rates among task conditions for the lifting and turning task and the moving a chair and desk together task [24, 25]. The level for significance was set at 0.05.

## Results

### Physical characteristics

Table 4 presents means and standard deviations of physical characteristics of participants, separated by gender and nationality. As a two-way ANOVA revealed no significant effects of gender or nationality, data for all participants were combined in further analyses. For all participants (N = 42), age showed a significant positive correlation with height (r = 0.762, p < 0.01), weight (r = 0.547, p < 0.01), and grip strength (r = 0.535, p < 0.01).

**Table 4:** Physical characteristics of participants.

Participants	Indonesian		Japanese		All
	Boy (N = 6)	Girl (N = 11)	Boy (N = 12)	Girl (N = 13)	N = 42
	M ± SD	M ± SD	M ± SD	M ± SD	M ± SD
Age (years)	7.5 ± 1.0	7.6 ± 1.0	8.3 ± 0.8	8.3 ± 0.8	7.8 ± 1.0
Height (cm)	122.3 ± 4.6	122.7 ± 7.4	126.0 ± 6.0	126.0 ± 6.0	122.7 ± 7.1
Weight (kg)	25.6 ±5.1	23.8 ± 3.1	26.2 ± 3.1	26.2 ± 3.1	24.5 ± 3.5
BMI^a^ (kg/m2)	17.2 ± 2.58	15.9 ± 1.9	16.4 ± 1.2	16.4 ± 1.2	16.3 ± 1.7
Grip strength (kgf)	9.5 ±1.7	8.7 ± 1.6	10.0 ± 2.8	10.0 ± 2.8	9.23 ± 2.2

^a^Body mass index.

### Carrying a chair

**Fig 8:**
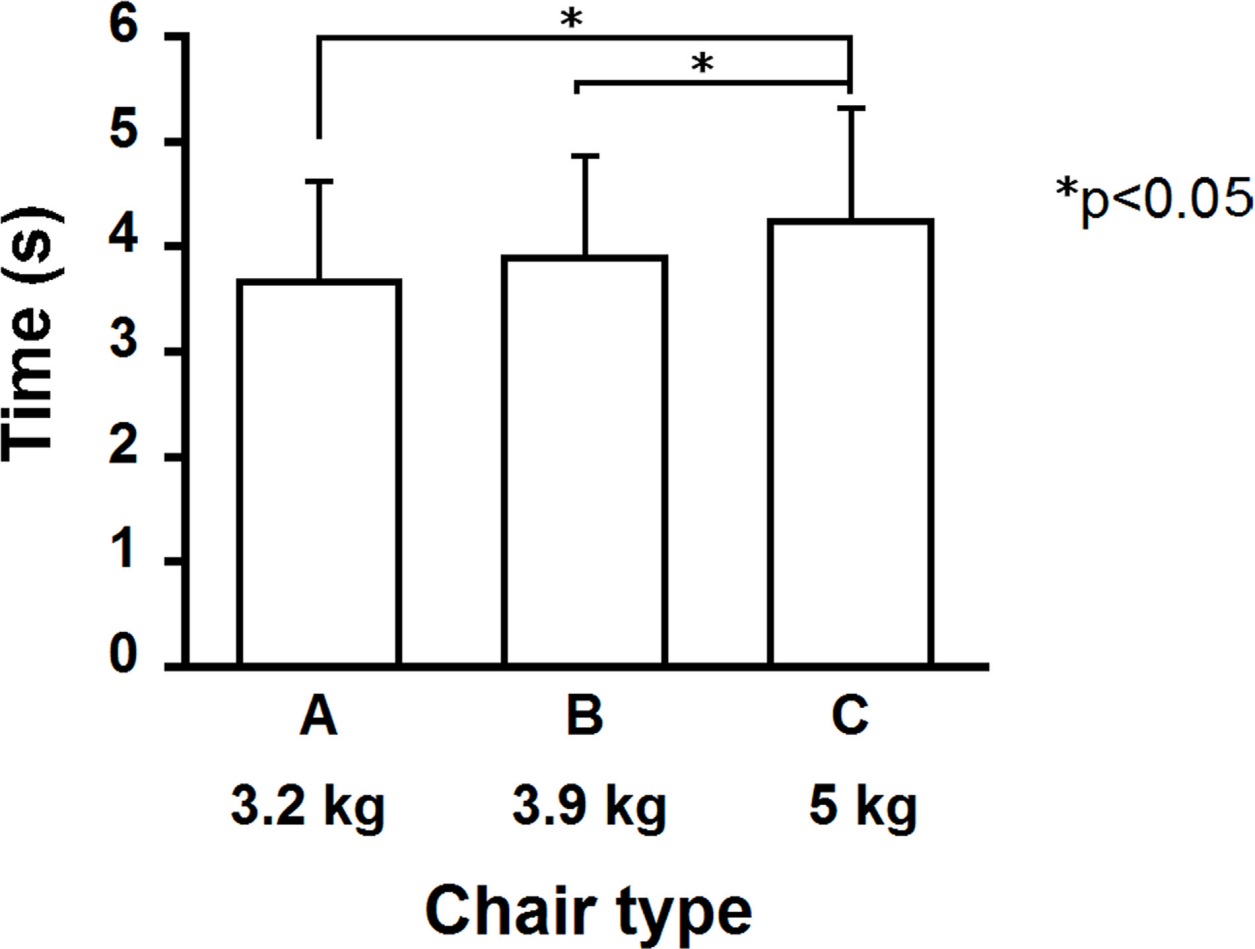
Task time for carrying only a chair.

**Fig 9:**
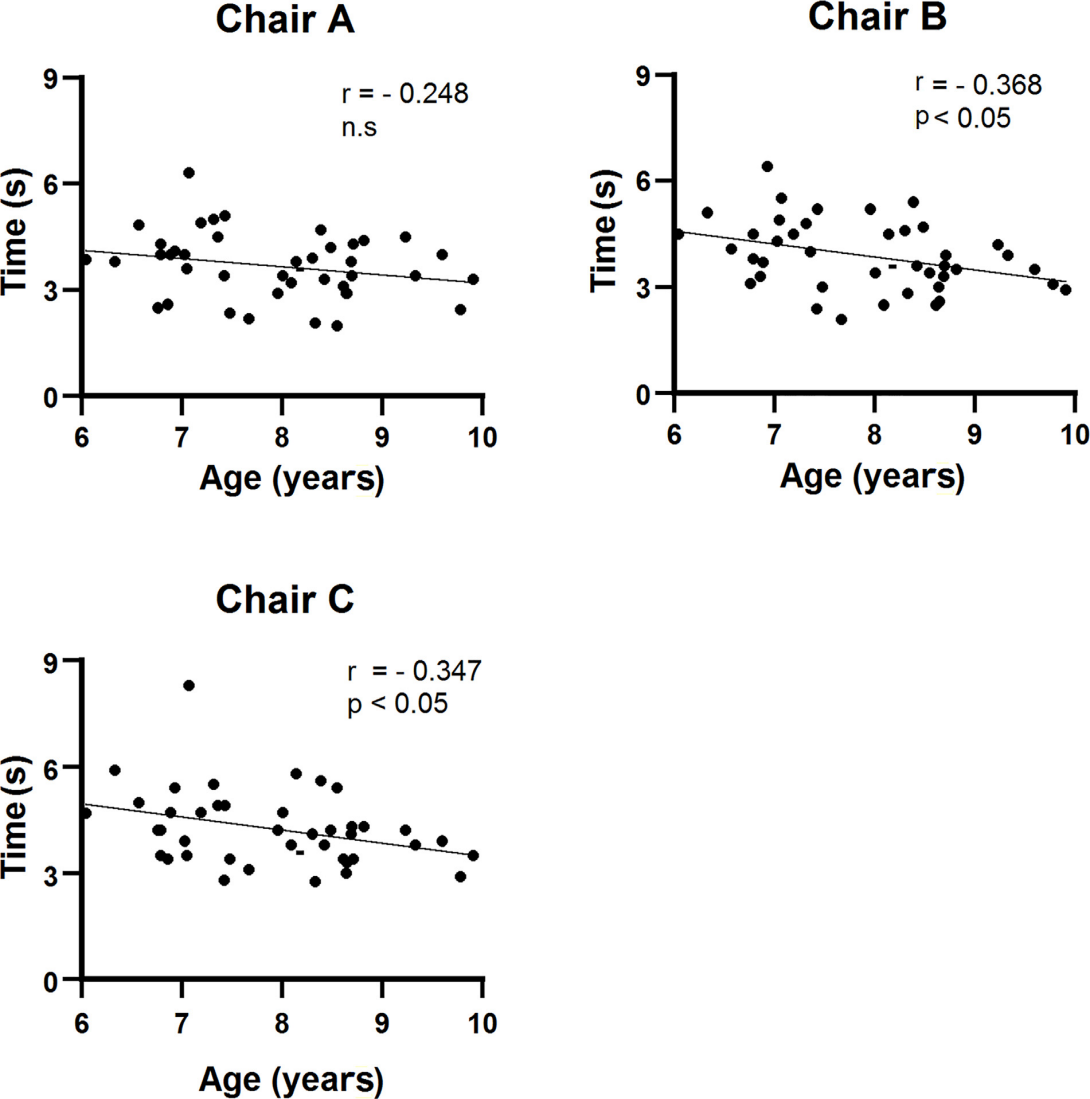
Relationship between participant age and task time for each chair condition.

All participants successfully completed the task of carrying a chair for all chair conditions. Fig 8 displays the task time for carrying only a chair a distance of three meters. The task time for Chair C was significantly longer than that of Chairs A and B (p < 0.05). A repeated measures one-way ANOVA showed a significant effect of chair type on task time (p < 0.01). Post-hoc tests using the Bonferroni correction revealed a significant difference between Chairs A and C, and between Chairs B and C (p < 0.05). Fig 9 illustrates the relationship between participant age and task time for each chair condition. There were significant negative relationships between these variables for Chair B (r = -0.368, p < 0.05) and C (r = -0.347, p < 0.05), but not for Chair A. However, task time was not significantly correlated with other physical characteristics (height, weight, BMI, and grip strength).

### Lifting and turning a chair

**Fig 10:**
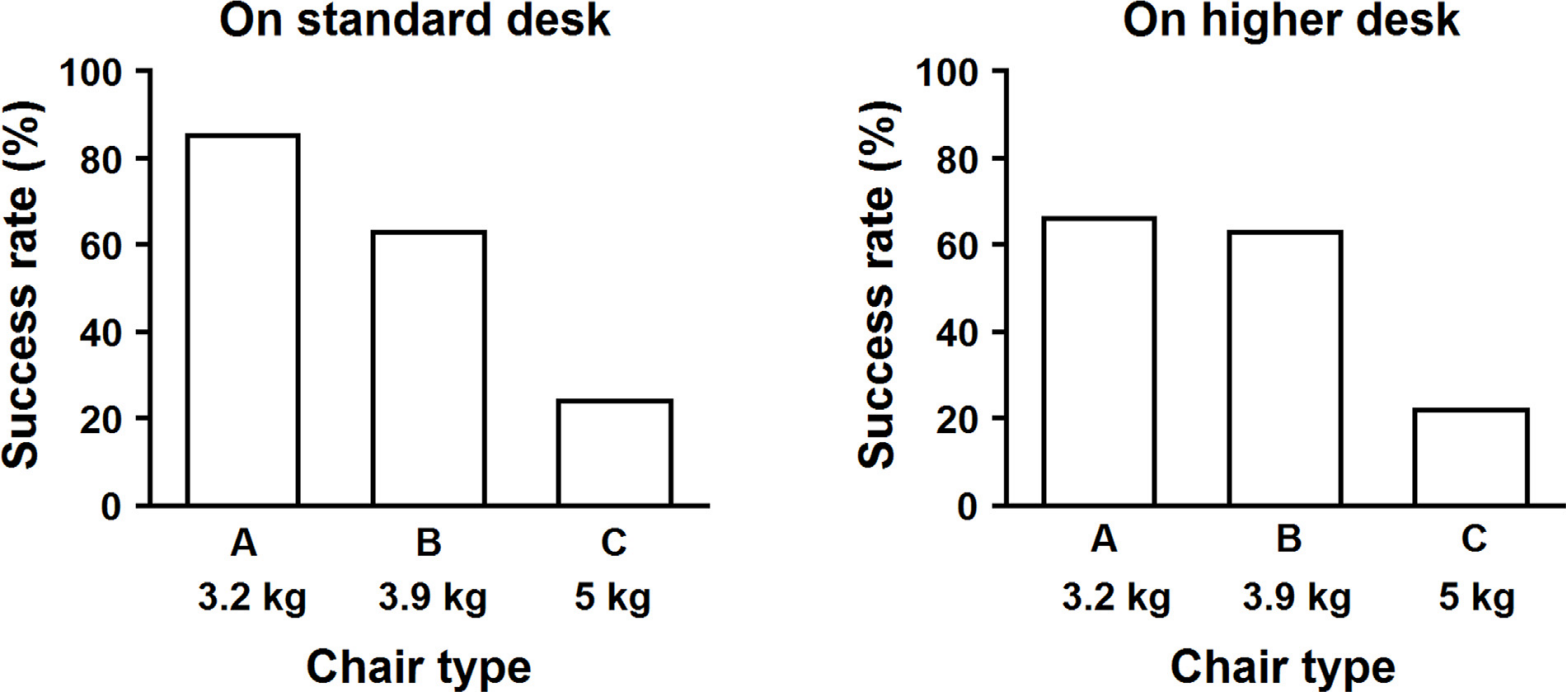
Success rates by chair type for lifting a chair on a standard/higher desk. Exact chi square test: standard desk: p < 0.01; higher desk: p < 0.01.

In both desk height conditions, the exact chi-square test revealed significant differences in success rates of lifting and turning a chair among the three chair conditions (p < 0.01). Chairs A and B showed success rates over 60%, whereas Chair C showed a lower success rate (around 20%) for both desk height conditions (Fig 10). In contrast, a significant effect of desk height on success rate was not found for any chair condition.

**Fig 11:**
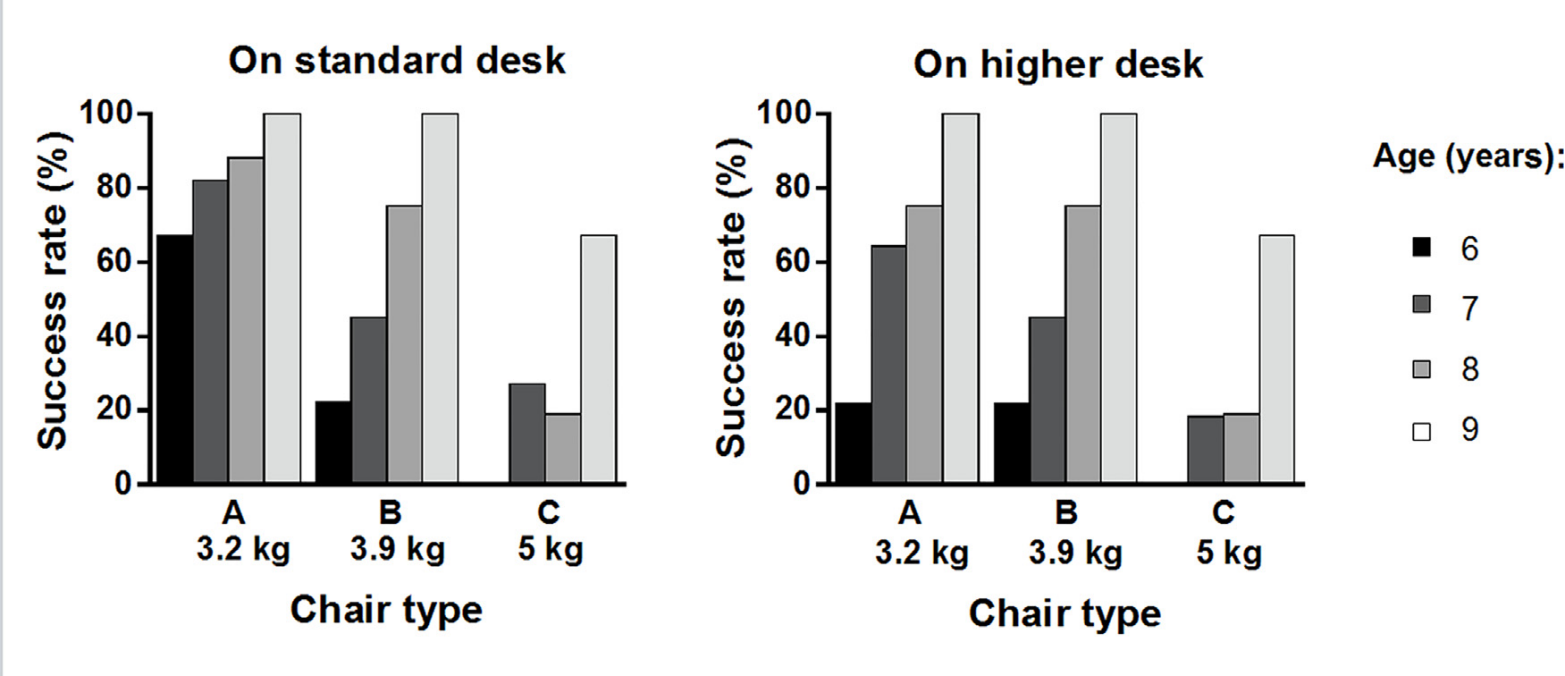
Success rates for chair type by age for lifting a chair on a standard/higher desk. Exact chi square test among chair types. Standard desk: age 6, p < 0.01; age 7, p = 0.051; age 8, p < 0.01; age 9, n.s. Higher desk: age 6, n.s; age 7, n.s; age 8, p < 0.01; age 9, n.s.

Fig 11 presents the effects of chair type and age on success rates for turning and lifting a chair in both desk height conditions. Success rates increased with age for all combinations of chair type and desk height, except for Chair C on a standard height desk. Participants aged 9 years attained a success rate of 100% for Chairs A and B in both desk height conditions. For participants aged 8 years, the success rate was significantly affected by chair type for both desk heights. Approximately 80% succeeded with Chairs A and B, but success rates dropped dramatically to approximately 20% for Chair C. For participants aged 7 in both desk height conditions, success rates gradually decreased from Chair A, to B, and to C, although the effect was of borderline significance in the standard height condition (p = 0.051). In addition, the success rate of participants aged 6 years showed a significant effect of chair type only for a standard desk. Although a high success rate (approximately 60%) was shown for Chair A and a standard desk height, very low rates were found for other combinations of chair types and desk heights.

### Carrying both a chair and a desk

**Fig 12:**
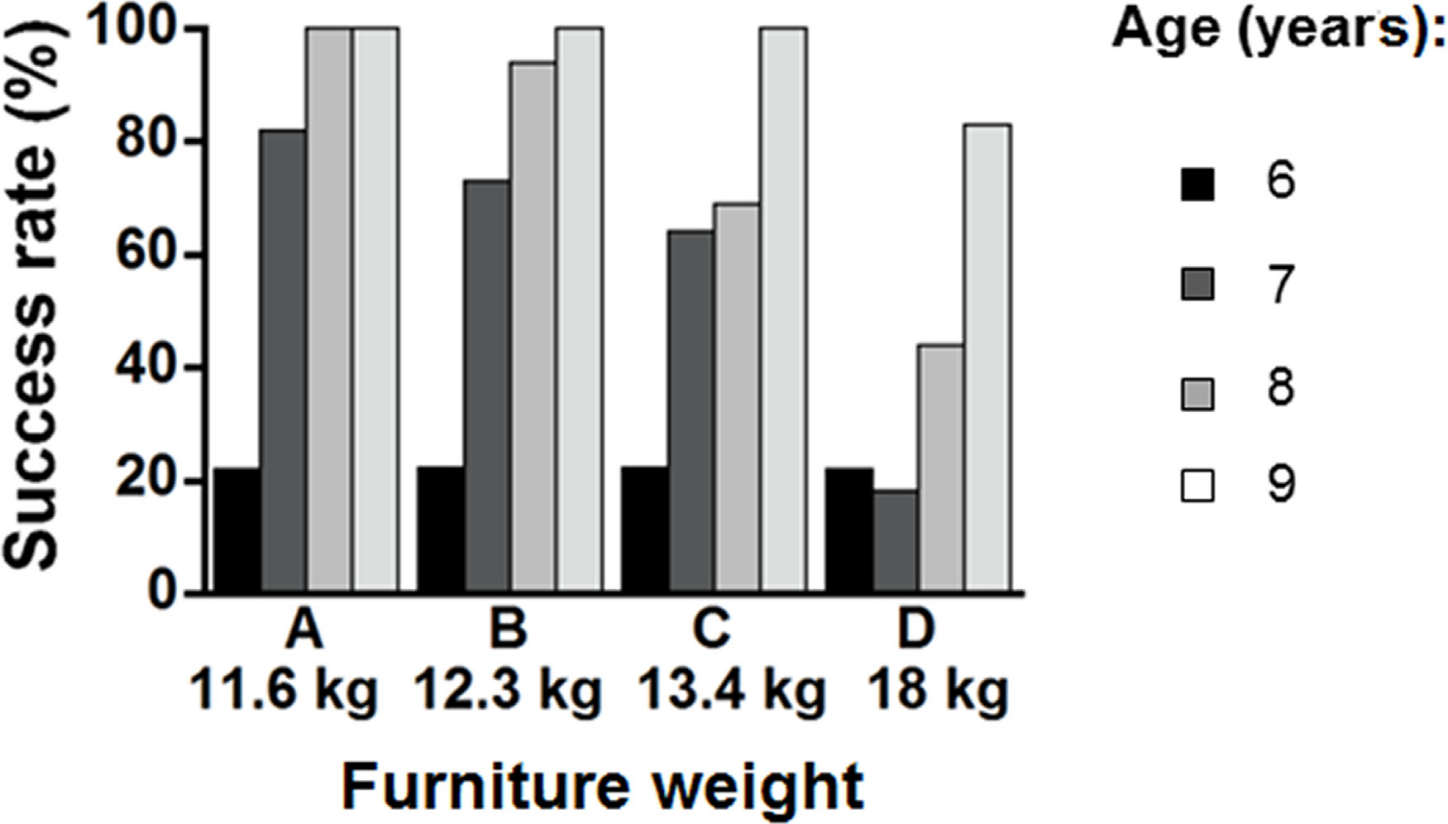
Success rates by four furniture weights for carrying a chair/desk together. Exact chi square test: p < 0.01.

**Fig 13:**
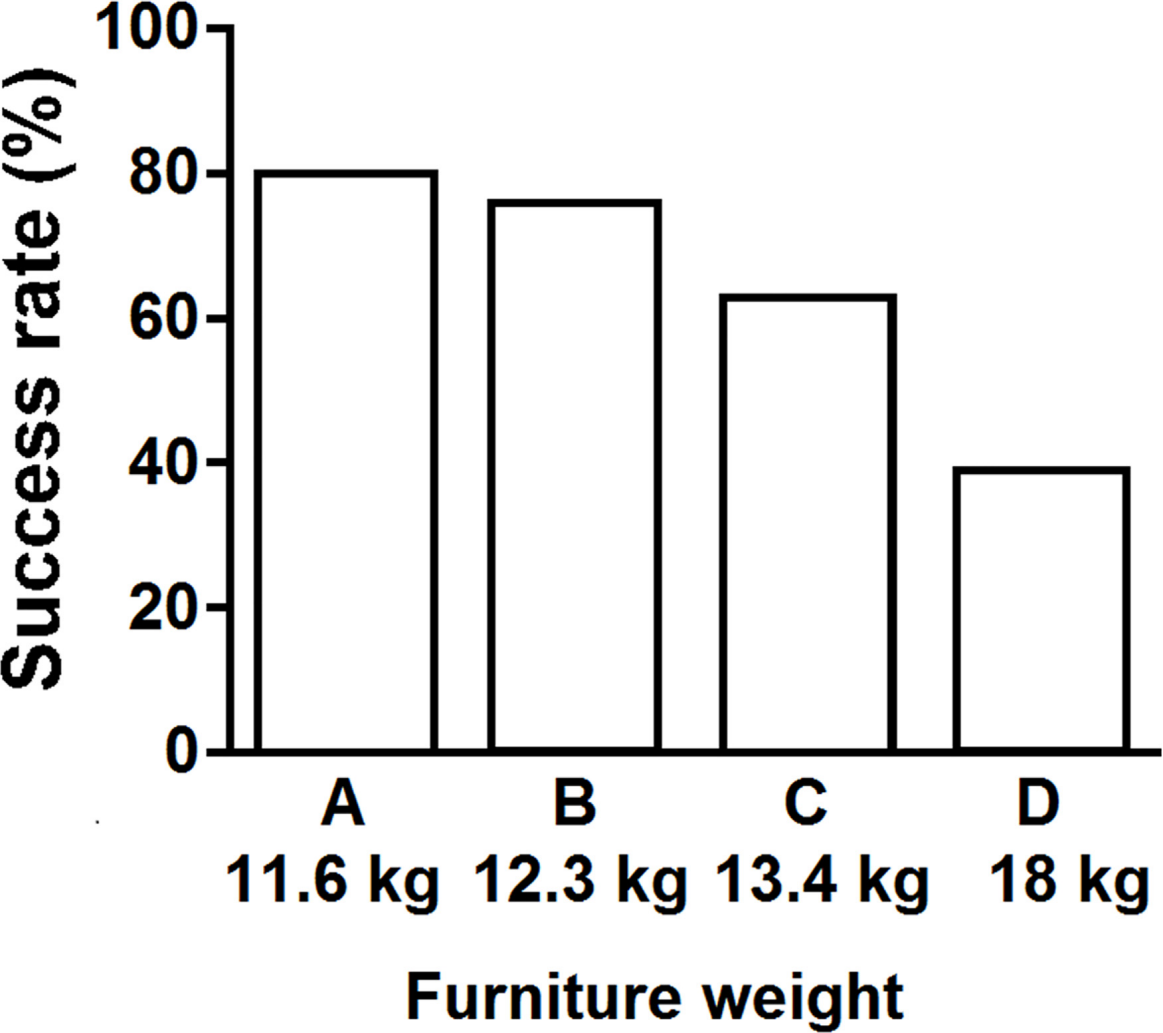
Success rates for four furniture weights by age group for carrying a chair/desk together. Exact chi square test: age 6, n.s.; age 7, p < 0.05; age 8, p < 0.05; age 9, n.s.

For the task that involved carrying both a chair and a desk, the exact chi-square test showed a significant effect of task condition on success rate (p < 0.01). Success rate decreased with increasing total weight of the chair and desk (Fig 12). Fig 13 presents the effects of task condition and age on success rate. For participants aged 7 and 8, the exact chi-square test also indicated significant differences in the rate among task conditions (p < 0.05). Success rates decreased with increasing total weight of the desk and chair, although success rates were nearly 100% for combinations A and B in children aged 8. However, this trend was not found for participants aged 6 and 9. For participants aged 9, success rates higher than 80% were observed for all conditions. In contrast, the success rate for participants aged 6 was lower (approximately 20%) for all conditions.

## Discussion

### Effects of gender and ethnic groups on physical measurements

The present study found no significant differences in physical measurements between genders. In previous research, gender differences have been regarded as an important factor in designing the size of elementary school furniture [26]. However, other studies of elementary school furniture have demonstrated that gender is not an important factor in determining appropriate dimensions of school furniture [27, 28]. Furthermore, boys and girls younger than age 10 have no significant differences in grip strength [29, 30]. Given that children in this experiment were less than 10 years old, we did not separate participants by gender.

This study compared Japanese and Indonesian chairs, used in their respective countries, and involved children of Japanese and Indonesian ethnicity. Given that Japanese children are familiar with Japanese chairs, they would be expected to perform better with Japanese chairs; the same would be true for Indonesian children and Indonesian chairs. Therefore, to ensure equality, this study included children who were of Japanese and Indonesian ethnicity. Previous research has found significant effects of ethnic group on anthropological measurements in children [31, 32]. Ethnicity, geography, and social conditions have been shown to influence physical characteristics [32, 33, 34]. However, the present study did not demonstrate significant differences for any measurements of physical characteristics between Indonesian and Japanese children. Moreover, according to the national anthropological database of Japanese and Indonesian individuals [23, 35, 36], heights and weights of children aged 6 to 9 are similar for the two groups. Therefore, Japanese and Indonesian children were grouped in the present analyses.

### Carrying a chair

Chairs are frequently carried for class activities in elementary schools. In the present study, the task time of Chair C was significantly higher than for the other two chairs (Fig 8). The prominent features of Chair C are larger dimensions and a heavier weight. Our previous study [37] using the same three types of chairs observed holding positions for carrying chairs, and found two popular positions. The differences in height of the popular holding positions for Chairs A and C are 34% (height of seat back lower rail: Chair A, 410 mm; Chair C, 550 mm) and 36% (seat height: Chair A, 300 mm; Chair C, 410 mm). In contrast, the difference in chair weight is 56% (Chair A, 3.2 kg; Chair C, 5.0 kg). Thus, the weight ratio is greater than the height ratio of the chairs’ holding positions. Accordingly, the longer task time—namely, the decrease in performance for Chair C, might be mainly caused by the heavier weight. This assumption is supported by findings [38–40] that heavy loads decreased walking speed of participants. Chair C is the typical type used in Indonesian public elementary schools. The heavy weight of this chair could potentially discourage dynamic class activities.

Furthermore, the present study showed a negative relationship between participant age and task time for Chairs B and C, but not Chair A (Fig 9). In other words, only Chair A was suitable for younger children to carry. Chair A is produced for children whose height is between 117 and 130 cm, in accordance with ISO 5970 [17]. This range corresponds to children aged approximately 6 to 7 years in both ethnic groups [23, 35]. ISO 5970 was created specifically so that children could be seated with proper posture. Our results suggest that an easy-to-carry chair can be produced for younger children within the guidelines of ISO 5970 [17].

On the other hand, some studies recommend that the weight of carried items should be proportional to children’s body weight [5–7]. In addition, the body weight of children normally increases with age [8–10]. In the present study, however, only age (and not body weight) had a significant relationship with task time. Age is comprehensive in that it involves aspects beyond physical characteristics (height, weight, physical strength), such as comprehension, skill, experience. Therefore, age is considered the best indicator for chair carrying specifications.

### Turning a chair upside down and desk height

Chairs are sometimes lifted and turned upside down in elementary schools in some countries [41, 42]. When students clean their classroom, this task is useful to create free floor space. In order to improve the efficiency of class activities, chairs and desks are often carried together by students to the perimeter of the room, or to corridors outside the room. In Japanese elementary schools, before carrying the chairs and desks, students usually lift and turn the chair upside down on the desk. Ease of this task is affected by not only the type of chair, but also the height of the desk. Therefore, the present study focused on the effects of these two aspects of school furniture, as well as the characteristics of participants.

The success rate for this task was strongly influenced by participant age (Fig 11) for both desk height conditions. Children aged 6 showed a much lower success rate for all types of chairs, compared with other older children. Moreover, even for the lightest chair (Chair A), they did not show a high success rate for either lower or higher desks (67% and 22%). Based on observations of their behavior during the task, most cases of failure seemed to be caused by not only physical characteristics (e.g., strength to lift the chair, high desk height compared to the child’s height) but also insufficient understanding of how to perform the task. This is in line with Rebok et al. [43], who demonstrated that comprehension of instructions among elementary school children declines with decreasing age. Accordingly, our findings suggest that in order to prevent injuries among children aged 6, they need appropriate instruction and supervision when lifting and turning even a lighter chair onto a desk.

On the other hand, among children aged 7 to 9, success rates depended on chair type as well as age. All children aged 9 succeeded at the task for Chairs A and B, but some did not for Chair C. These failures were mainly due to strength limitations, unlike for children aged 6. Children’s muscle strength increases with age [30], which is consistent with the grip strength results of the present study. Nevertheless, the 5.0 kg weight of Chair C was still too heavy even for some children aged 9. For children aged 7 to 8, success rate decreased as chair weight increased and as age decreased, in both desk height conditions, except for Chair C on a standard desk.

The present study failed to show significant effects of desk height on success rates for lifting and turning a chair. Some previous studies have demonstrated an effect of destination height when humans manually lift a load from the floor [44, 45]. Thus, it is not that the desk height had no effect on the ease of this task, but rather that the effect of the chair’s characteristics, such as size and weight, exceeded that of the desk height.

### Carrying a chair and desk together

In the current study, the success rate of carrying a chair and desk together decreased as age decreased, and as total weight of the chair and desk increased (Fig 13). Furniture Combination D simulated an Indonesian chair and desk. Children’s success rate in the 6–9 year age range was much lower than in other conditions (Fig 13). In a previous study of adults lifting an object to hand height in a standing straight position once during eight hours, the ratios between the maximum acceptable weight and the body weight of males and females were 34.1% and 25.7%, respectively [46]. However, in the present study, the ratio of the Combination D weight (18 kg) to children’s body weight (mean: 24.5 kg) was 73.5%. To our knowledge, there are no studies investigating maximum acceptable carrying weights for children. However, the weight ratio of Combination D to children’s average body weight in the study was more than twice the acceptable weight ratio for adult. Therefore, the weight of Indonesian school furniture should be decreased to make it more suitable for carrying.

Children aged 6 showed a very low success rate for this task (approximately 22%), which was much different from those aged 7 and over (Fig 13). As described, the previous task (lifting and turning a chair) was also difficult for them. Accordingly, these results indicate that carrying a chair and desk together is not recommended for younger children. Meanwhile, the success rate of children aged 7 to 8 decreased with increased weight of furniture. In children aged 8, the success rate was close to 100% at and below 12.3 kg of total weight (Combination B). The chair and desk in Combination A and B are mainly produced for children of heights corresponding to those aged 6–7 and 8–9, respectively, and meet the guideline of ISO 5970 [17] (e.g., children aged 7 using Combination A, children aged 8 and 9 using Combination B) were very high. It is expected that this is a familiar task that is often performed in Japanese elementary schools. Therefore, these findings suggest that guidelines for furniture weights for this task should be provided separately according to the age of the child.

### Implications, limitation and future research priorities

The present study provided useful information regarding weight guidelines of elementary school furniture for promoting classroom activities (Table 5). However, it is not possible to propose ideal weights for each age and task, because only a few participants of each age were included in this study, and only three types of chairs were tested. Future research should aim to develop an optimal threshold for elementary school chair weights, in order to extend the practical applications of these findings.

**Table 5:** Weight guidelines of elementary school chairs and desks for children aged 6–9.

Arrangement activities	Weight guidelines
Carrying a chair	Although a chair weight of 5.0 kg can be carried, lighter chairs enable easier carrying.
	For children aged 6 to 7, chair weights at or under 3.2 kg are preferable.
Lifting and turning a chair	Lighter chairs enable the task to be performed easily. However, appropriate instruction and supervision are required for all chair types, especially for children aged 6, even if the chair is light enough.
	A 5.0 kg chair weight is too heavy for all children.
Carrying a chair and desk	This task is not recommended for younger children, especially those aged 6, because of safety considerations.
	For children aged 8, a total weight of furniture at or below 12.3 kg is preferable.
	A total weight of 18.0 kg (simulated Indonesian furniture) is too heavy for all children.

The findings of the present study support the need for lighter chairs to encourage more dynamic class activities. However, decreasing the weight of furniture, while still manufacturing durable and strong products, may be difficult and unaffordable for developing countries. Frail chairs may lead to injuries due to lack of quality control with regard to safety. Therefore, future research could alternatively focus on the key parts of the chair, which are usually held by children, as an effective alternative strategy to improve ease of chair transport.

## Conclusions

The findings of the present study indicate that children’s carrying of a chair, carrying both a chair and a desk, and lifting and turning a chair onto a desk, are strongly influenced by not only children’s age, but also features of the chair, especially weight. Based on the findings of the present study, the weight guideline of furnitures for elementary school children aged 6–9 was proposed (Table 5). Decreasing the weight of Indonesian elementary school furniture that is too heavy for children (especially children of younger ages) is recommended to encourage dynamic class activities. School furniture size and weight should be appropriate for children’s physical characteristics. Consideration given to furniture size and weight will help to enhance children’s classroom participation and improve classroom activities, thereby leading to better quality education.

## References

[1] Marx A, Fuhrer U, Hartig T. Effects of classroom seating arrangements on children's question-asking. Learning Environments Research. 1999; 2: 249–263.

[2] Wannarka R, Ruhl K. Seating arrangements that promote positive academic and behavioural outcomes: a review of empirical research. Support for Learning. 2008; 23: 89–93.

[3] Haghighi MM, Jusan MM. Exploring students behavior on seating arrangements in learning environment: a review. Procedia-Social and Behavioral Sciences. 2012; 36: 287–294.

[4] Breithecker D. Workplace school demands on ergonomic school furniture for today’s classroom; 2007. E-print. Available: http://www.lsfurnishings.com/pdfs/VS/%2BBAG7_Educational_Workplace_long_US.pdf. Accessed 18 October 2014.

[5] Bridger R. Introduction to ergonomics. London: CRC Press; 2008.

[6] Sander M. Weight of schoolbags in a Freiburg elementary school. Recommendations to parents and teachers (author's transl)]. Das Offentliche Gesundheitswesen. 1979; 251–253. 156328

[7] Hong Y, Brueggemann GP. Changes in gait patterns in 10-year-old boys with increasing loads when walking on a treadmill. Gait and Posture. 2000; 11: 254–259. 10802438 10.1016/s0966-6362(00)00055-2

[8] Li JX, Hong Y, Robinson PD. The effect of load carriage on movement kinematics and respiratory parameters in children during walking. European Journal of Applied Physiology. 2003; 90: 35–43. 12783230 10.1007/s00421-003-0848-9

[9] Waterlow JC, Buzina R, Keller W, Lane JM, Nichaman MZ, Tanner JM. The presentation and use of height and weight data for comparing the nutritional status of groups of children under the age of 10 years. Bulletin of the World Health Organization.1977; 55: 489 304391 PMC2366685

[10] Tanner JM, Whitehouse RH, Takaishi M. Standards from birth to maturity for height, weight, height velocity, and weight velocity: British children, 1965. I. Archives of Disease in Childhood. 1966; 4: 454–471. 10.1136/adc.41.219.454 PMC2019592 5957718

[11] O'Loughlin J, Gray-Donald K, Paradis G, Meshefedjian G. One-and two-year predictors of excess weight gain among elementary school children in multi ethnic, low-income, inner-city neighborhoods. American Journal of Epidemiology.2000; 152: 739–746. 11052551 10.1093/aje/152.8.739

[12] Feathers D, Pavlovic-Veselinovic S, Hedge A. Measures of fit and discomfort for elementary school children in Serbia. Work: Journal of Prevention, Assessment and Rehabilitation.2013; 44: 73–81. 10.3233/WOR-2012-01563 23241689

[13] Castellucci HI, Arezes PM, Viviani CA. Mismatch between classroom furniture and anthropometric measures in Chilean schools. Applied Ergonomics. 2010; 41: 563–568. 10.1016/j.apergo.2009.12.001 20031115

[14] Saarni L, Nygård CH, Kaukiainen A, Rimpelä A. Are the desks and chairs at school appropriate?. Ergonomics. 2007; 50: 1561–1570. 17917897 10.1080/00140130701587368

[15] Domljan D, GrbacI, Hadina J. Classroom furniture design—correlation of pupil and chair dimensions. Collegium Antropologicum. 2008; 32: 257–265. 18494212

[16] Geldhof E, De Clercq D, De Bourdeaudhuij I, Cardon G. Classroom postures of 8–12 year old children. Ergonomics. 2007; 50: 1571–1581. 17917898 10.1080/00140130701587251

[17] ISO5970. Furnitures—Chairs and tables for educational institutions—functional sizes. Geneva, Switzerland: International Organization for Standardization; 1979 10.1684/abc.2008.0205

[18] Ministry of National Education of Republic of Indonesia. Regulation of Ministry of National Education of Republic of Indonesia no. 24 year 2007 about standard of facilities and infrastructure for elementary school/madrasah ibtidaiyah (SD/MI), junior high school / madrasah tsanawiyah (SMP/MTS), and high school/madrasah tsanawiyah (SMA/MA). Jakarta: Ministry of National Education of Republic of Indonesia; 2007.

[19] Purwaningrum L, Aryani SM, Mulyadi, YassierlieThe product design evaluation of elementary school furniture. Proceeding of the 4th International Product Design and Development in conjunction with the 4th AUN/SEED-Net Regional Conference of Manufacture. Yogyakarta, Indonesia; 2011.

[20] Ministry of national education. Regulation of Ministry of National Education of Republic of Indonesia no. 32 year 2011, standard and technical specification of rebuilding broken classroom, construction of new classroom and furnitures, and construction of library and furniture for elementary school/elementary school of children with disabilities. Jakarta: Ministry of National Education of Republic of Indonesia; 2011.

[21] SNI 7555.19. Wood and wood products-part 19: Learning to primary schools chairs, ICS 97.140. Jakarta: BSN (Badan Standardisasi Nasional); 2011.

[22] Yosita L. Preliminary study to primary education facilities: A comparison study between Indonesia and development countries. Dimensi Teknik Arsitektur. 2006; 34: 122–132.

[23] Rosyidi CN, Susmartini S, Purwaningrum L, Muraki S. Mismatch analysis of elementary school desk and chair key characteristics in Indonesia. Applied Mechanics and Materials. 2014; 660: 1057–1061.

[24] Field A. Discovering statistics using IBM SPSS statistics. London: Sage; 2013.

[25] Sheskin DJ. Handbook of parametric and nonparametric statistical procedures. USA: Crc Press; 2004.

[26] Jeong BY, Park KS. Sex differences in anthropometry for school furniture design. Ergonomics. 1990; 33: 1511–1521. 2286197 10.1080/00140139008925350

[27] Dhara PC, Khaspuri G, Sau SK. Complaints arising from a mismatch between school furniture and anthropometric measurements of rural secondary school children during classwork. Environmental Health and Preventive Medicine. 2009; 14: 36–45. 10.1007/s12199-008-0055-8 19568866 PMC2684770

[28] Adewole NA, Isedowo B. Excel interface utilization in automation of design process of ergonomic classroom furniture for primary school pupils in nigeria. International Journal of Scientific and Engineering Research. 2012; 3: 388–396.

[29] Ross CH, Ro¨sblad B. Norms for grip strength in children aged 4–16 years. Acta Paediatrica. 2002; 91: 617–625. 12162590 10.1080/080352502760068990

[30] Beenakker EAC, Van der Hoeven JH, Fock JM, Maurits NM. Reference values of maximum isometric muscle force obtained in 270 children aged 4–16 years by hand-held dynamometry. Neuromuscular Disorders. 2001; 11: 441–446. 11404114 10.1016/s0960-8966(01)00193-6

[31] Cotton LM, O’Connell DG, Palmer PP, Rutland MD. Mismatch of school desks and chairs by ethnicity and grade level in middle school. Work. 2002; 18: 269–280 12441567

[32] Chuan TK, Hartono M, Kumar N. Anthropometry of the Singaporean and Indonesian populations. International Journal of Industrial Ergonomics. 2010; 40: 757–766

[33] Habicht JP, Yarbrough C, Martorell R, Malina RM, Klein RE. Height and weight standards for preschool children: How relevant are ethnic differences in growth potential?. Lancet. 1974; 303: 611–615. 10.1016/s0140-6736(74)92663-4 4132271

[34] Graitcer PL, Gentry E. Measuring children: one reference for all. Lancet. 1981; 318: 297–299. 10.1016/s0140-6736(81)90538-9 6114335

[35] Ministry of Education, Culture, Sports, Science and Technology [MEXT) of Japan. Physical fitness and motor ability investigation (transleted); 12 October 2014. Database [internet]. Accessed: http://www.e-stat.go.jp/SG1/estat/NewList.do?tid=000001016672

[36] Ministry of Internal Affairs and Communications Statistics Bureau of Japan. Physical fitness and motor ability investigation in 2013[translated]; 12 October 2014. Database [internet]. Available: http://www.e-stat.go.jp/SG1/estat/List.do?bid=000001055014&cycode=0.

[37] Purwaningrum L, Muraki S. The observation of children’s holding position to redesign elementary school chair for easy carrying and moving. Proceeding of 1st Asian Conference on Ergonomics & Design 2014. Jeju: South Korea; 2014.

[38] Kinoshita H. Effects of different loads and carrying systems on selected biomechanical parameters describing walking gait. Ergonomics. 1985; 28: 1347–1362. 4065090 10.1080/00140138508963251

[39] Nottrodt JW, Manley P. Acceptable loads and locomotor patterns selected in different carriage methods. Ergonomics. 1989; 32: 945–957. 2806225 10.1080/00140138908966856

[40] LaFiandra M, Wagenaar RC, Holt KG, Obusek JP. How do load carriage and walking speed influence trunk coordination and stride parameters?. Journal of Biomechanics. 2003; 36: 87–95. 4 12485642 10.1016/s0021-9290(02)00243-9

[41] Ministry of Education, Culture, Sports, Science and Technology of Japan. Guidebook for Starting School; April 2005. E-print. Available: http://www.mext.go.jp/component/english/__icsFiles/afieldfile/2011/03/17/1303764_008.pdf. Accessed 22 October 2014.

[42] Anderson M. What Every 4th Grade Teacher Needs to Know About Setting Up and Running a Classroom, Chapter One: Classroom Setup; 2010. Preprint. Available: https://www.responsiveclassroom.org/sites/default/files/et4ch1.pdf. Accessed: 22 October 2014.

[43] Rebok G, Ri ley A, Forrest C, Starfield B, Green B, Robertson J, Tambor E. Elementary school-aged children's reports of their health: a cognitive interviewing study. Quality of Life Research. 2001; 10: 59–70. 11508476 10.1023/a:1016693417166

[44] National Institute of Occupational Safety and Health (NIOSH). Work practices guide for manual lifting, NIOSH technical report no.81-122 US Department of Health and Human Services. Cincinnati, Ohio: National Institute for Occupational Safety and Health; 1981.

[45] Waters TR, Putz-Anderson V, Garg A, Fine LJ. Revised NIOSH equation for the design and evaluation of manual lifting tasks. Ergonomics. 1993; 36: 749–776. 8339717 10.1080/00140139308967940

[46] Snook SH, Ciriello VM. The design of manual handling tasks: revised tables of maximum acceptable weights and forces. Ergonomics. 1991; 34: 1197–1213. 1743178 10.1080/00140139108964855

